# Association between hemostatic changes and contusion volume in traumatic brain injury: an observational cohort study

**DOI:** 10.1007/s00701-026-06768-9

**Published:** 2026-01-17

**Authors:** Alexander Fletcher-Sandersjöö, Emma Hammarlund, Caroline Lindblad, Logan Froese, Henrike Häbel, Jennifer Sebghati, Marc Maegele, Mikael Svensson, Bo-Michael Bellander, David W. Nelson, Eric Peter Thelin

**Affiliations:** 1 Department of Clinical Neuroscience, Karolinska Institutet, Bioclinicum J5:20, 171 64 Solna, Stockholm Sweden; 2 Department of Neurosurgery, Karolinska University Hospital, Stockholm, Sweden; 3 Function Perioperative Care and Medicine, Karolinska University Hospital, Stockholm, Sweden; 4 Department of Medical Sciences, Acquired Brain Injury, Uppsala University, Uppsala, Sweden; 5 Biomedical Engineering, Faculty of Engineering, University of Manitoba, Winnipeg, Canada; 6 Department of Learning, Informatics, Management and Ethics, Karolinska Institutet, Stockholm, Sweden; 7 Department for Trauma and Orthopedic Surgery, Cologne-Merheim Medical Centre, University Witten/Herdecke, Cologne, Germany; 8 Institute for Research in Operative Medicine, University Witten/Herdecke, Cologne, Germany; 9 Department of Physiology and Pharmacology, Karolinska Institutet, Stockholm, Sweden; 10 Medical Unit Neurology, Karolinska University Hospital, Stockholm, Sweden

**Keywords:** Traumatic brain injury, Coagulopathy, Hemostasis, Contusion volume, Contusion expansion, Hematoma volume

## Abstract

**Purpose:**

Contusion expansion is a key determinant of outcome after traumatic brain injury (TBI). Because many patients develop acute coagulopathy, it has been proposed that hemostatic changes may drive this expansion, but the link remains uncertain.

**Methods:**

In this retrospective single-center cohort, we included adults with isolated moderate-to-severe TBI and no pre-injury antithrombotic therapy. The hemostatic markers activated partial thromboplastin time (APTT), prothrombin time (PT, reported as INR), platelet count (PLT), and fibrinogen were measured on admission and during the first 72 h. Contusion volumes were derived from serial CT scans. Associations between hemostatic markers and contusion volumes over time were analyzed using generalized additive mixed models (GAMMs), adjusting for confounders.

**Results:**

Among 109 patients, median admission values were fibrinogen 2.4 g/L, PT-INR 1.0, APTT 29 s, and PLT 233 × 10^9^/L. After admission, fibrinogen and PLT declined, whereas PT-INR and APTT increased modestly. Contusion volume increased from a median of 0.7 ml at baseline to 4.6 ml on the third CT. In univariable models, higher APTT and PT-INR values and lower platelet counts were associated with larger contusion volumes, but these associations lost significance after adjustment for age and time from injury.

**Conclusion:**

Hemostatic disturbances, as measured by standard coagulation assays, were common after TBI but not independently associated with contusion volume over time.

**Supplementary Information:**

The online version contains supplementary material available at 10.1007/s00701-026-06768-9.

## Introduction

Outcomes after traumatic brain injury (TBI) are shaped not only by the primary insult but also by secondary processes that worsen the injury. Among these, contusion expansion stands out as both common and strongly linked to poor prognosis, while also offering a potential therapeutic target [1, 10, 12]. However, clinical trials of hemostatic agents have so far failed to limit hematoma expansion [7], leaving uncertainty about which components of the post-traumatic hemostatic response are most relevant to intervene upon.

TBI-induced coagulopathy includes hyperfibrinolysis [2, 6, 13, 19, 23, 25, 26, 30, 33], consumption of clotting factors [3, 13, 15, 17, 22, 25–27, 29], and reductions in both platelet count (PLT) [25] and function [16, 21]. Abnormalities present at admission have been associated with hematoma expansion [6, 32, 39], but much less is known about how coagulation markers evolve after admission – the period when interventions can be applied. To date, only one study has suggested that a decline in platelet count (PLT) during the first 24 h may contribute to expansion, whereas other markers showed no clear association [5].

To address this knowledge gap, we aimed to examine the temporal course of routine hemostatic markers after isolated moderate-to-severe TBI, and their relationship with contusion volume. Our aim was to clarify whether evolving hemostatic changes contribute to contusion expansion and whether standard laboratory assays provide clinically relevant information for guiding therapy.

## Methods

### Study design and population

We conducted a single-center retrospective cohort study at the Karolinska University Hospital, the sole level 1 trauma center for a catchment area of approximately 2.4 million people. Adult patients (≥ 15 years) admitted between 2006 and 2021 with isolated moderate-to-severe TBI, defined as a Glasgow Coma Score [31] (GCS) of 3–13 on admission, were eligible.

Exclusion criteria were penetrating injury, multitrauma (extracranial Abbreviated Injury Scale [14] [AIS] ≥ 3), pre-injury antithrombotic therapy, absence of a cerebral contusion, uncertain injury time, initial CT performed > 12 h after injury, no follow-up CT within 72 h, or contusion evacuation before follow-up imaging. To ensure longitudinal data, patients also needed measurements of activated partial thromboplastin time (APTT), prothrombin time (PT, reported as INR), PLT, and fibrinogen taken at or before the first CT and repeated after the second CT. The 72-h window was chosen because most traumatic contusions stabilize within this period [10], making it a clinically meaningful timeframe for assessing contusion expansion and hemostatic changes.

The study was approved by the Swedish Ethical Review Authority who waived the need for informed consent.

### Data collection

Patients were identified from a local trauma database that records all TBI admissions. Clinical data were obtained from electronic medical records using the TakeCare (CompuGroup Medical Sweden AB) and Clinisoft (Centricity Critical Care, GE Healthcare) software, while imaging data were retrieved from Sectra PACS (Sectra AB).

Collected variables included demographics, comorbidities, injury characteristics, neurological status, hemostatic markers (APTT, PT-INR, PLT, fibrinogen), CT findings, surgical treatment, and 12-month Glasgow Outcome Scale [18] (GOS). Hemostatic markers were routinely sampled at admission and daily thereafter, with additional testing as clinically indicated. GOS was assessed by research nurses through validated questionnaires and structured telephone interviews. Patients were managed according to institutional protocols, including monitoring and correction of coagulation abnormalities. Data on hemostatic interventions (e.g., tranexamic acid, fibrinogen concentrate, platelet transfusion) were not included in the analysis, as these treatments were often recorded in handwritten notes or given on verbal orders without consistent documentation, making it unreliable for systematic extraction. The study period did not overlap with the introduction of evidence supporting tranexamic acid, and this therapy was therefore not routinely administered.

### Contusion volume calculations

CT imaging was performed at admission, typically repeated at 6 and 24 h, and thereafter as clinically indicated (see Appendix A for full scan distribution). Contusion volumes were calculated using semi-automated computer-assisted volumetric analysis, as previously described [10, 34], with manual review and adjustment before rounding to the nearest 0.1 ml. For patients with multiple contusions, volumes were summed.

### Statistical analyses

All data curation and analyses were performed in R (R Development Core Team, http://www.R-project.org/; R Foundation for Statistical Computing, Vienna, Austria) with RStudio®. The primary outcome was absolute contusion volume at each CT time point, and the primary exposures were APTT, PT-INR, PLT, and fibrinogen. Linear interpolation (zoo package [40]) was used to align the time series of hemostatic markers with the contusion volume measurements, providing matched values for each data point as illustrated in Appendix B. We considered linear interpolation appropriate as these variables typically change gradually over 12–24 h. Temporal changes in hemostatic markers were visualized with ggplot2 [36], and corrplot [35] visualized inter-variable relationships. However, because linear interpolation may smooth short-lived physiological fluctuations, we also performed a sensitivity analysis using a nearest-neighbor approach. For each CT time point, the closest available laboratory value was substituted using last-observation-carried-forward or last-observation-carried-backward, depending on temporal proximity. Univariable and multivariable models were re-estimated to assess robustness.

We employed generalized additive mixed models GAMMs (mgcv package [38]) to model relationships between contusion volume and hemostatic markers, allowing for non-linear relationships via smooth functions [37] employing restricted maximum likelihood estimation. Random effects accounted for between-patient heterogeneity, and an autocorrelation structure (corAR1) addressed non-independence in longitudinal data. A gamma distribution with a log link was used to handle right-skewed data and reduce the influence of non-linear variables in the model. See Appendix C for ACF/PACF plots.

After selecting the best random-effects structure based on the Akaike Information Criterion (AIC), we performed univariable analyses with contusion volume as the dependent variable and APTT, PT-INR, PLT, fibrinogen, sex, time from injury, age, GCS, and pupil response as independent variables. Continuous variables were modeled with cubic splines, and categorical/ordinal variables were linear. All significant variables in univariable analyses were then included in a stepwise backward multivariable model, retaining only those remaining significant.

## Results

### Study population

**Fig. 1:**
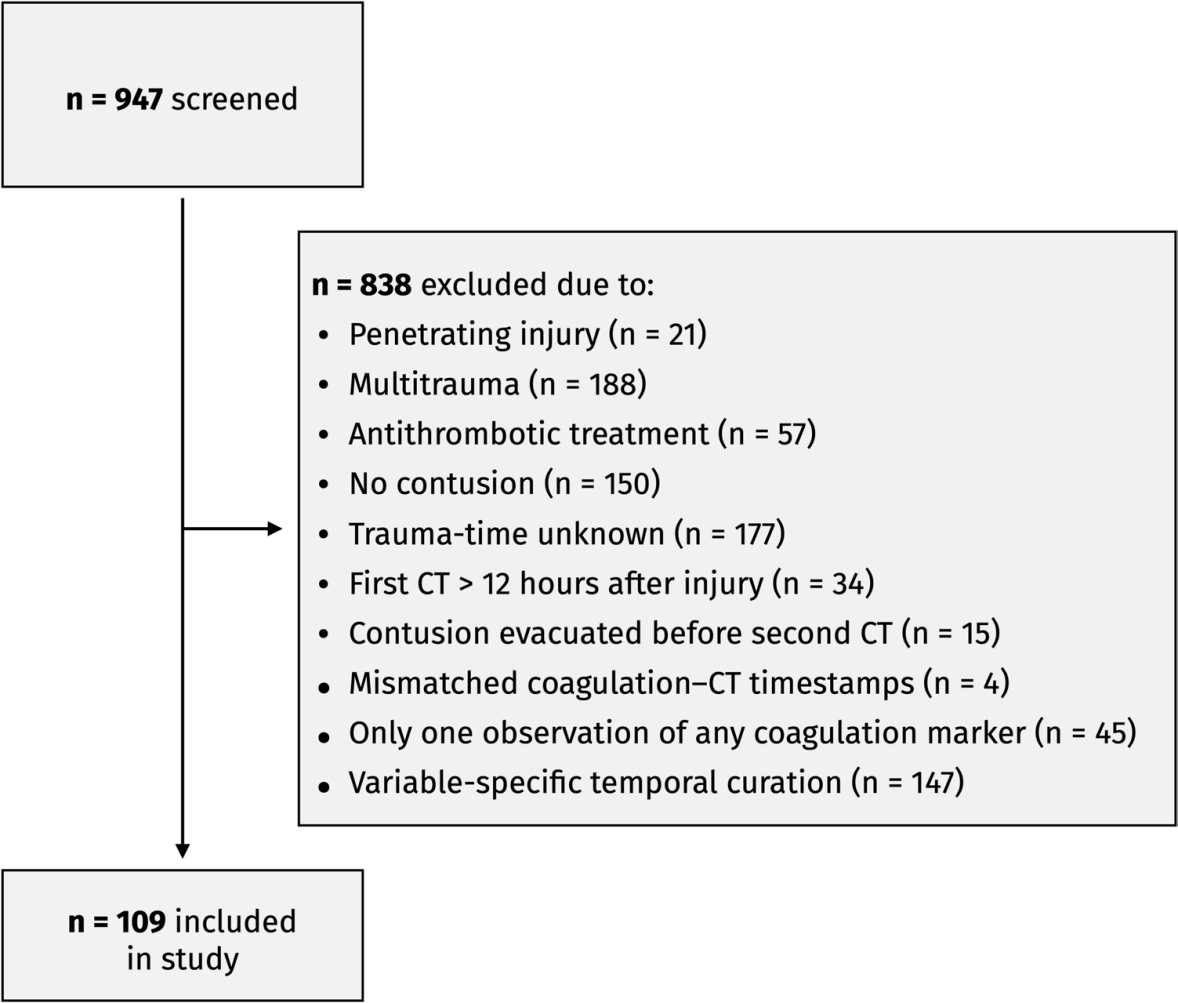
Flow chart of the patient inclusion process

A total of 947 adult patients with moderate-to-severe TBI were screened. Of these, 838 were excluded for the following reasons: penetrating injury (*n* = 21), multitrauma with extracranial AIS ≥ 3 (*n* = 188), pre-injury antithrombotic therapy (n = 57), absence of a cerebral contusion (n = 150), unknown trauma time (n = 177), first CT performed > 12 h after injury (n = 34), contusion evacuation before follow-up CT (*n* = 15), mismatched coagulation–CT timestamps (*n* = 4), having only a single measurement of any coagulation marker (n = 45), and not meeting the longitudinal laboratory–imaging requirements described in the Methods (n = 147) (Fig. 1). The final study cohort therefore comprised 109 patients who fulfilled all imaging and laboratory criteria.

The cohort was predominantly male (76%), with a median age of 43 years (IQR 27–57) and a median admission GCS of 7 (IQR 4–9). Median admission fibrinogen was 2.4 g/L, PT-INR 1.0, APTT 29 s, and PLT 233 × 10^9^/L. Median contusion volume increased from 0.7 ml (IQR: 0.2–1.5) at baseline to 2.0 ml (IQR: 0.4–9.0) on the second CT and 4.6 ml (IQR: 0.8–9.0) on the third CT (Table 1).

**Table 1:** Characteristics of the study cohort

Variable	All patients (*n* = 109)
Age (years)	43 (27–57)
Male sex	83 (76%)
GCS on admission	7 (4–9)
Unilateral dilated unresponsive pupil	13 (12%)
Bilateral dilated unresponsive pupil	14 (13%)
Labs on admission	
APTT (seconds)	29 (26–32)
PT-INR	1.0 (1.0–1.1)
Fibrinogen (g/L)	2.4 (2.0–2.9)
PLT (× 10^^9^/L)	233 (194–271)
Marshall CT Classification on admission	
Class I	0 (0%)
Class II	40 (37%)
Class III	6 (6%)
Class IV	7 (6%)
Class V-VI	56 (51%)
Time to CT 1 (hours)	1.4 (1.1–1.6)
Time to CT 2 (hours)	7.7 (5.5–9.9)
Time to CT 3 (hours)	35 (20–67)
CT1 contusion volume (ml)	0.7 (0.2–1.5)
CT2 contusion volume (ml)	2.0 (0.4–9.0)
CT3 contusion volume (ml)	4.6 (0.8–9.0)
Treatment	
Intubated	104 (95%)
Invasive neuromonitoring	87 (80%)
Craniotomy	56 (51%)
12-month GOS	4 (3–5)
Unfavorable GOS at 12-months	44 (40%)

Data shown as median (interquartile range) or number (proportion). Abbreviations: *APTT*  activated partial thromboplastin time, *CT* Computed tomography, *g* grams, *GCS* Glasgow Coma Scale, *GOS* Glasgow Outcome Scale, *INR* international normalized ratio, *L* liters, *PLT* platelet count, *PT* prothrombin time

### Time course of hemostatic markers

**Fig. 2:**
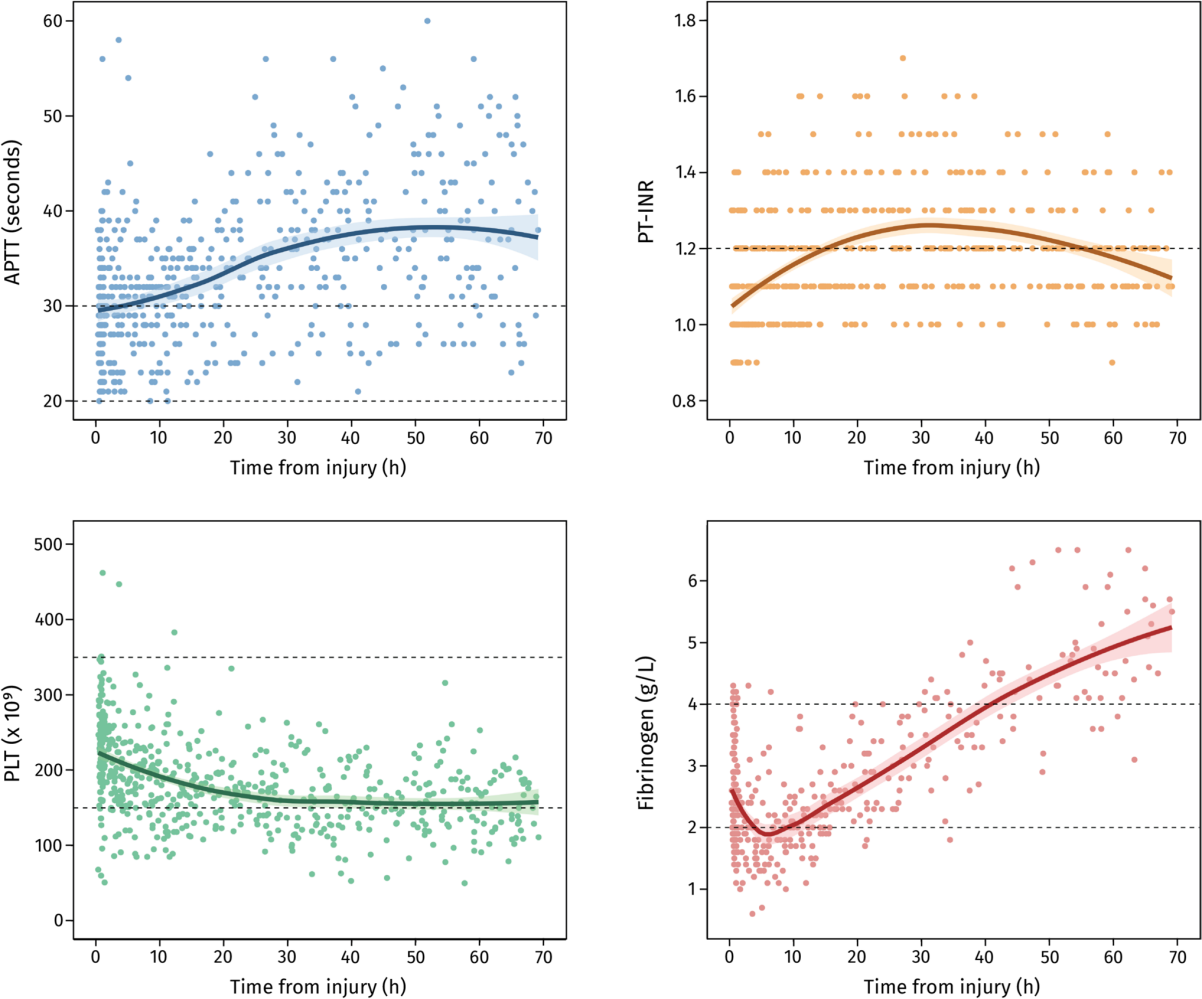
Hemostatic markers over time following traumatic brain injury. Each point represents an individual measurement. LOESS curves (with 95% confidence intervals as shaded areas) illustrate overall trends. Dashed black lines denote normal reference ranges. Abbreviations: APTT = activated partial thromboplastin time; g = grams; h = hours; INR = international normalized ratio; L = liters; PLT = platelet count; PT = prothrombin time

On admission, median values were within reference ranges: fibrinogen 2.4 g/L, PLT 233 × 10^9^/L, PT-INR 1.0, and APTT 29 s. Distinct trajectories emerged thereafter (Fig. 2). Fibrinogen levels fell sharply during the first 10 h, consistent with early consumption, before rising again, likely reflecting recovery and acute-phase reactivity. Platelet counts declined steadily for about 30 h, though most values remained above 150 × 10^9^/L. PT-INR rose modestly, peaking at just over 1.1 between 20 and 30 h, while APTT increased progressively, with many patients exceeding the upper reference limit after 24 h.

Taken together, these patterns suggest early depletion of clotting factors and platelets with mild prolongation of coagulation times. However, most results stayed near normal ranges, making their clinical relevance uncertain.

### Association between hemostatic markers and contusion volumes

Before GAMM analysis, a correlation plot revealed moderate negative correlations between PLT and PT-INR (r = –0.38, p = 0.049) and between PLT and time from trauma (r = –0.32, p = 0.05) (Appendix D), but these were not deemed strong enough to motivate adjustment in the multivariable model. Comparing random effects structures indicated that the random intercept-only model (AIC = 864) provided a better fit than the model with both random intercept and slope (AIC = 875).

**Fig. 3:**
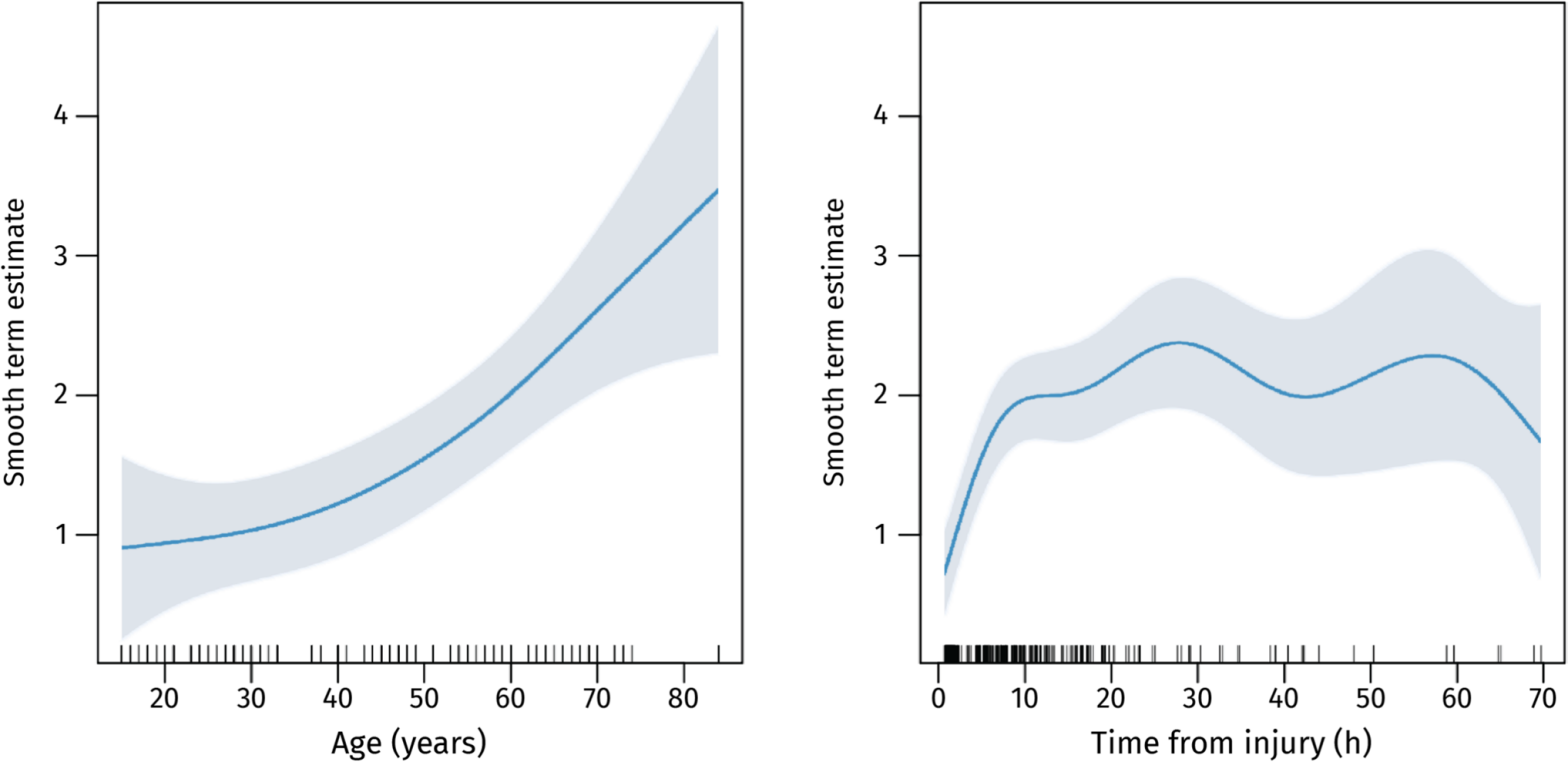
Smooth effects of age and time from trauma on contusion volume as modeled by the GAMM. The y-axis shows the estimated smooth effect for each variable, with LOESS curves and 95% confidence intervals (shaded areas) illustrating trends. Tick marks along the x-axis represent individual data points

**Fig. 4:**
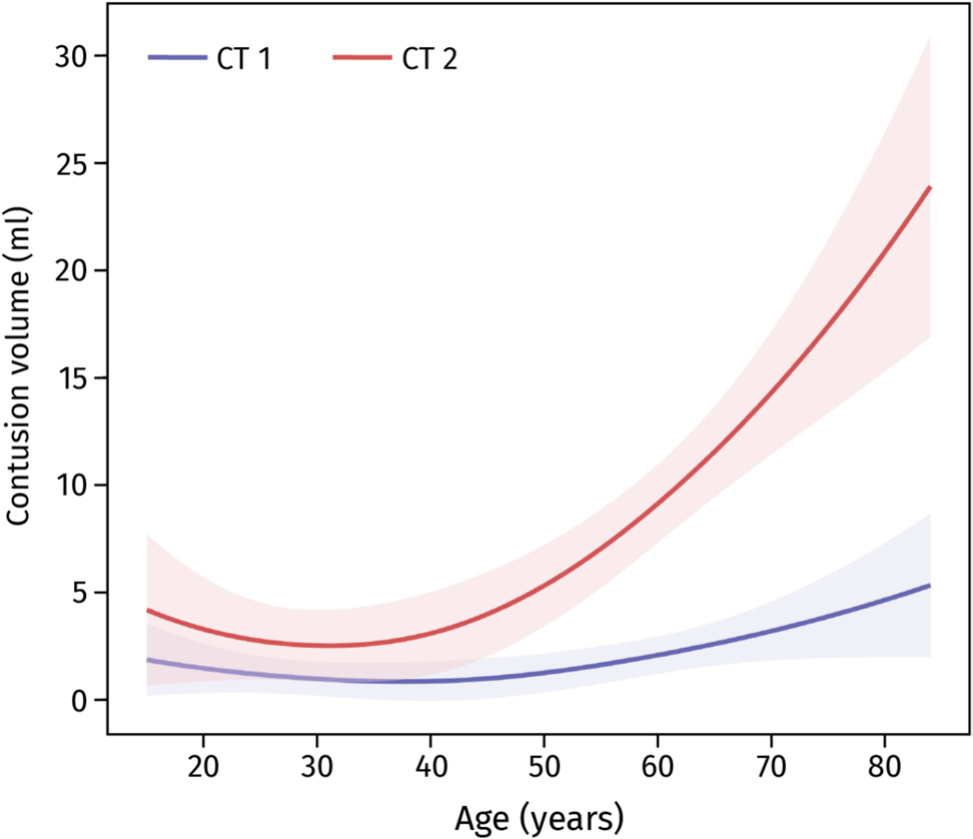
Relationship between age and contusion volume for the first and second CT scans. LOESS curves and 95% confidence intervals (shaded areas) depict the trend. Abbreviations: CT = computed tomography; ml = milliliters

In univariable models, higher APTT (*p* = 0.045) and PT-INR (*p* = 0.004) and lower PLT (*p* = 0.013) were associated with larger contusion volumes, while fibrinogen showed no significant association (Table 2). These findings suggested that patients with prolonged coagulation times or lower PLT tended to develop larger contusions. However, after adjustment for age and time from injury, none of the hemostatic markers remained independently associated with contusion volume. In the final multivariable model, only age (p < 0.001) and time from injury (*p* < 0.001) were retained (adjusted R^2^ = 0.18). Older patients had larger contusions across time points, and expansion was most pronounced between up to 20 h post-injury before plateauing (Figs. 3–4).

**Table 2:** Univariable and multivariable GAMM analysis of each covariate against contusion volume

	**Univariable model**			**Final step-down multivariable model**		
**Continuous covariates**	**edf**	**F-value**	**p-value**	**edf**	**F-value**	**p-value**
Age	1.00	11.1	** < 0.001**	1.71	13.8	** < 0.001**
Time from injury	6.47	27.7	** < 0.001**	6.36	27.9	** < 0.001**
APTT	1.00	4.05	**0.045**	–	–	–
PLT	1.00	6.33	**0.013**	–	–	–
PT-INR	2.08	5.77	**0.004**	–	–	–
Fibrinogen	1.00	2.71	0.101	–	–	–
**Categorical and ordinal covariates**	**estimate**	**t-value**	**p-value**	**estimate**	**t-value**	**p-value**
Sex	−0.24	−0.85	0.395	–	–	–
GCS	0.03	0.74	0.462	–	–	–
Unilateral unresponsive pupil	−0.56	−1.51	0.133	–	–	–
Bilateral unresponsive pupils	0.10	0.27	0.787	–	–	–

Variables not included in the final multivariable model are indicated by a dash (–) in the multivariable columns. Abbreviations: *APTT* activated partial thromboplastin time, *edf* estimated degrees of freedom, *PT* prothrombin time, *INR* international normalized ratio, *PLT* platelet count, *GCS* Glasgow Coma Scale. Bold text in the p-value column indicates a statistically significant correlation (*p* < 0.05)

Sensitivity analyses using nearest-neighbor substitution produced results highly consistent with the interpolated dataset. In univariable models, INR and platelet count remained significant predictors, while APTT lost significance (p = 0.0788), and fibrinogen remained non-significant. The multivariable model retained the same structure as the primary analysis: neither INR nor platelet count remained independently associated with expansion after adjustment for age and time from trauma. Thus, the choice of interpolation method did not materially affect model behavior or study conclusions.

## Discussion

In this cohort of patients with isolated moderate-to-severe TBI, we found that fibrinogen and PLT declined after admission, while PT-INR and APTT increased modestly. Contusion expansion occurred mainly within the first 20 h and then plateaued. In univariable analyses, lower PLT and higher PT-INR and APTT were linked to larger contusion volumes, but these associations did not persist after adjusting for age and time from injury. Together, these findings indicate that although coagulation changes are common after TBI, routine assays do not independently predict contusion expansion.

### Interpretation of coagulation markers

The admission fibrinogen level in our cohort (median 2.4 g/L) closely matches prior reports in TBI, where median values ranged from 2.4 to 2.5 g/L [23, 25]. After admission, we observed an early decline, likely reflecting factor consumption and fibrinolysis. Similar patterns have been reported elsewhere, with elevated D-dimer and fibrin degradation products supporting early fibrinolytic activation [6, 13, 19, 25, 26, 30, 33]. The subsequent rebound may be explained by inhibition of fibrinolysis [20, 24], administration of fibrinogen concentrate, or fibrinogen’s acute-phase nature as a hepatic reactant. PLT declined steadily during the first 30 h, consistent with previous descriptions of post-traumatic platelet consumption and dilution [11]. In most patients, levels remained above the reference range (> 150 × 10^9^/L), indicating that the changes may not have been severe enough to impair clot formation. Lastly, coagulation times showed only minor prolongations. PT-INR rose from 1.0 at admission to a peak of just over 1.1 at 20–30 h post-injury, while APTT increased gradually to 30–40 s. These changes also align with earlier studies reporting small but significant prolongations of PT and APTT after TBI [17, 22, 25].

Overall, our findings support the concept of a mild consumptive coagulopathy, detectable by standard assays but generally within or just outside reference ranges.

### Relationship with contusion expansion

In univariable models, lower PLT and higher PT-INR and APTT were associated with larger contusion volumes, while fibrinogen showed no association. These findings echo earlier studies linking admission PLT, PT, and fibrinolytic activity to progressive hemorrhagic injury [6, 32, 39]. However, none of the markers remained significant after adjusting for age and time from injury. Several explanations for this are possible. First, most values in our cohort were within reference ranges, suggesting that disturbances captured by standard assays may not have been severe enough to drive bleeding. Second, therapeutic interventions, such as fibrinogen concentrate, tranexamic acid, or platelet transfusions, may have corrected abnormalities, thereby obscuring associations. Third, APTT, PT-INR, platelet count, and fibrinogen may lack sensitivity for trauma-specific dysfunction [28], including impaired platelet function, altered thrombin generation, or changes in fibrinolysis. Finally, adjustment for age and time from injury may have removed important sources of biological variation. Older patients have been shown to exhibit greater coagulant activation and factor consumption after trauma [4], while time from injury strongly influences both laboratory values and contusion expansion.

### Study limitations and implications

As a retrospective observational study, our findings are primarily subject to treatment and selection bias as well as several confounders. Documentation of hemostatic interventions such as tranexamic acid, fibrinogen concentrate, and platelet transfusions was inconsistent, often confined to handwritten notes based on bedside verbal orders without standardized timestamps or dosing details. These variables were therefore excluded to avoid misclassification bias, which may have limited the ability to detect associations between hemostatic abnormalities and contusion expansion. Serial viscoelastic and platelet function data were not available for a sufficient number of patients and could therefore not be included. Consequently, the analyses relied on routine coagulation assays, which only partially capture trauma-related hemostatic disturbances and may overlook dynamic dysfunctions such as impaired platelet activation or fluctuations in fibrinolytic activity. As we were forced to exclude patients who had contusion evacuations, some of the more clinically relevant cases were not included in this analysis. These may also have constituted a bias towards younger patients in part explaining the contributing factor of older age to contusion expansion. Sampling was also limited, and interpolation between laboratory and imaging timepoints may have smoothed rapid fluctuations, although detailed sampling distributions and CT scan frequencies are presented in the Appendix A–B. The modest sample size and inclusion of patients with minor extracranial injuries (AIS 1–2) may also have influenced results. Finally, we did not assess the relationship between contusion expansion and clinical outcomes, as the cohort was underpowered for adjusted analyses and any positive findings would have been difficult to interpret causally. Instead, this association has previously been evaluated in this cohort in studies specifically designed for prognostic modelling, where absolute expansion demonstrated a clear dose–response relationship with 12-month GOS [8, 9]. Because those analyses were performed using the same underlying patient material, the present study focuses specifically on the mechanistic link between hemostatic markers and the time course of contusion growth rather than outcome prediction.

Despite these limitations, several implications emerge. Routine coagulation assays remain essential for identifying overt abnormalities, and on-admission tests may still be valuable for detecting systemic coagulopathy or guiding initial correction. However, serial measurements provide limited insight into the risk of contusion expansion. Clinically, this suggests that standard coagulation tests are insufficient for identifying patients at risk of contusion expansion or for guiding targeted hemostatic therapy. Instead, more informative approaches may include viscoelastic testing, platelet function assays, and markers of thrombin generation and fibrinolysis. Future studies should focus on denser early sampling and the use of more sensitive assays to better capture dynamic hemostatic changes and clarify whether specific abnormalities drive contusion expansion and thus represent targets for intervention.

## Conclusion

In patients with isolated moderate-to-severe TBI, routine coagulation markers changed over time but were not independently associated with contusion volume once age and time from injury were considered. Further research using more sensitive assays, combined with more frequent sampling, is needed to determine whether post-traumatic coagulopathy plays a direct role in contusion expansion in TBI.

## Supplementary Information

Below is the link to the electronic supplementary material.

Supplementary file1 (DOCX 2588 KB)

## Data Availability

The datasets used and/or analyzed during the current study are available from the corresponding author on reasonable request.

## Abbreviations

AIC: Akaike Information Criterion
AIS: Abbreviated Injury Scale
APTT: Activated partial thromboplastin time
CT: Computed tomography
edf: Estimated degrees of freedom
GAMM: Generalized additive mixed model
GCS: Glasgow Coma Scale
GOS: Glasgow Outcome Scale
INR: International normalized ratio
IQR: Interquartile range
LOESS: Locally estimated scatterplot smoothing
PACS: Picture Archiving and Communication System
PLT: Platelet count
PT: Prothrombin time
TBI: Traumatic brain injury
