# Supplementary material for: Association between hemostatic changes and contusion volume in traumatic brain injury: an observational cohort study
Source: Acta Neurochir (Wien). 2026 Jan 17;168(1):14. doi: 10.1007/s00701-026-06768-9 (PMC12815991; doi:10.1007/s00701-026-06768-9)

**Appendix A:** Distribution of CT scans over time. Most patients underwent two or three CT scans within the first 72 hours post-injury. One patient underwent four CT scans during this period.


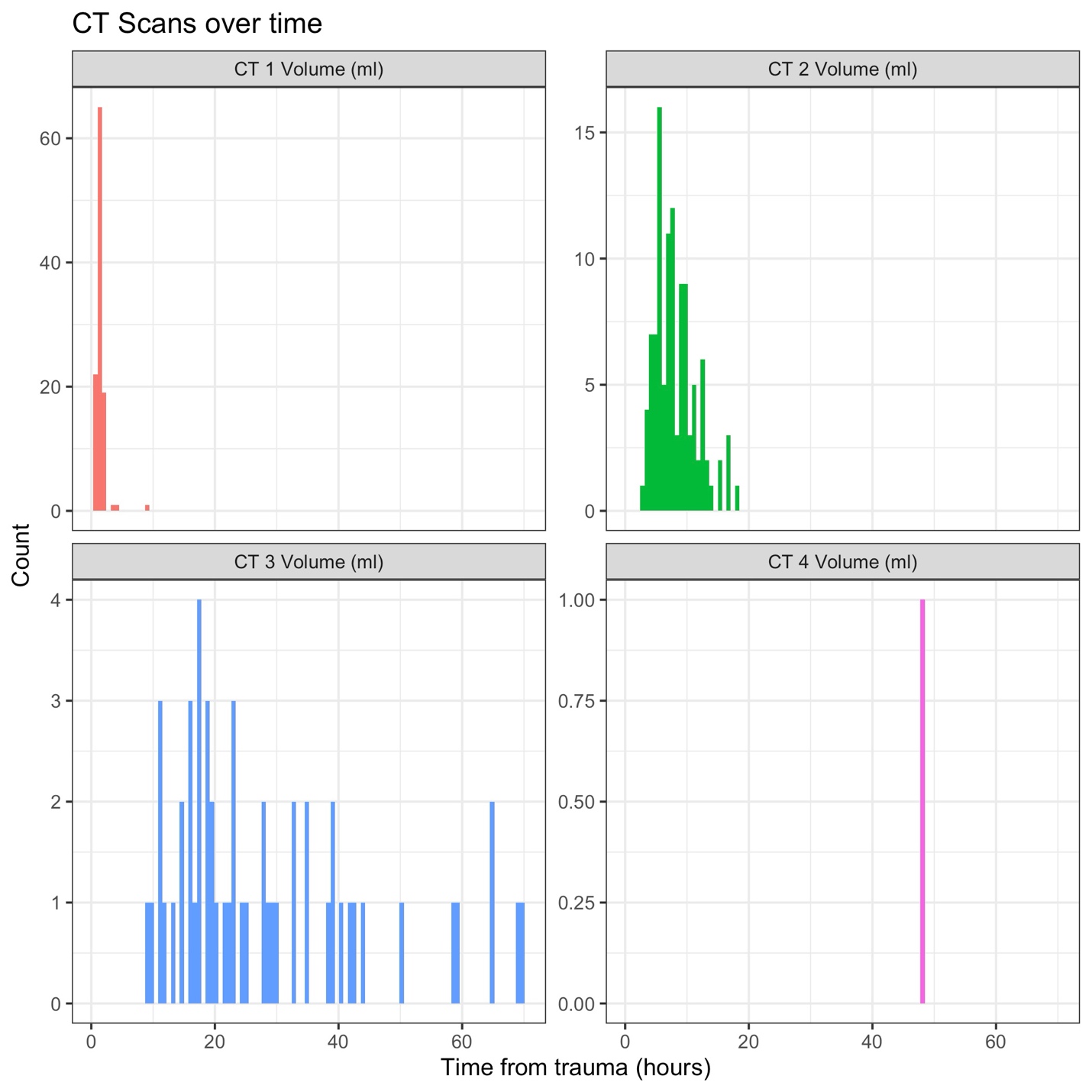


**Appendix B**: Distribution of hemostatic markers before (left) and after (right) linear interpolation. Hemostatic marker values were linearly interpolated to align with CT scan time points and create a consistent time series for analysis.

**
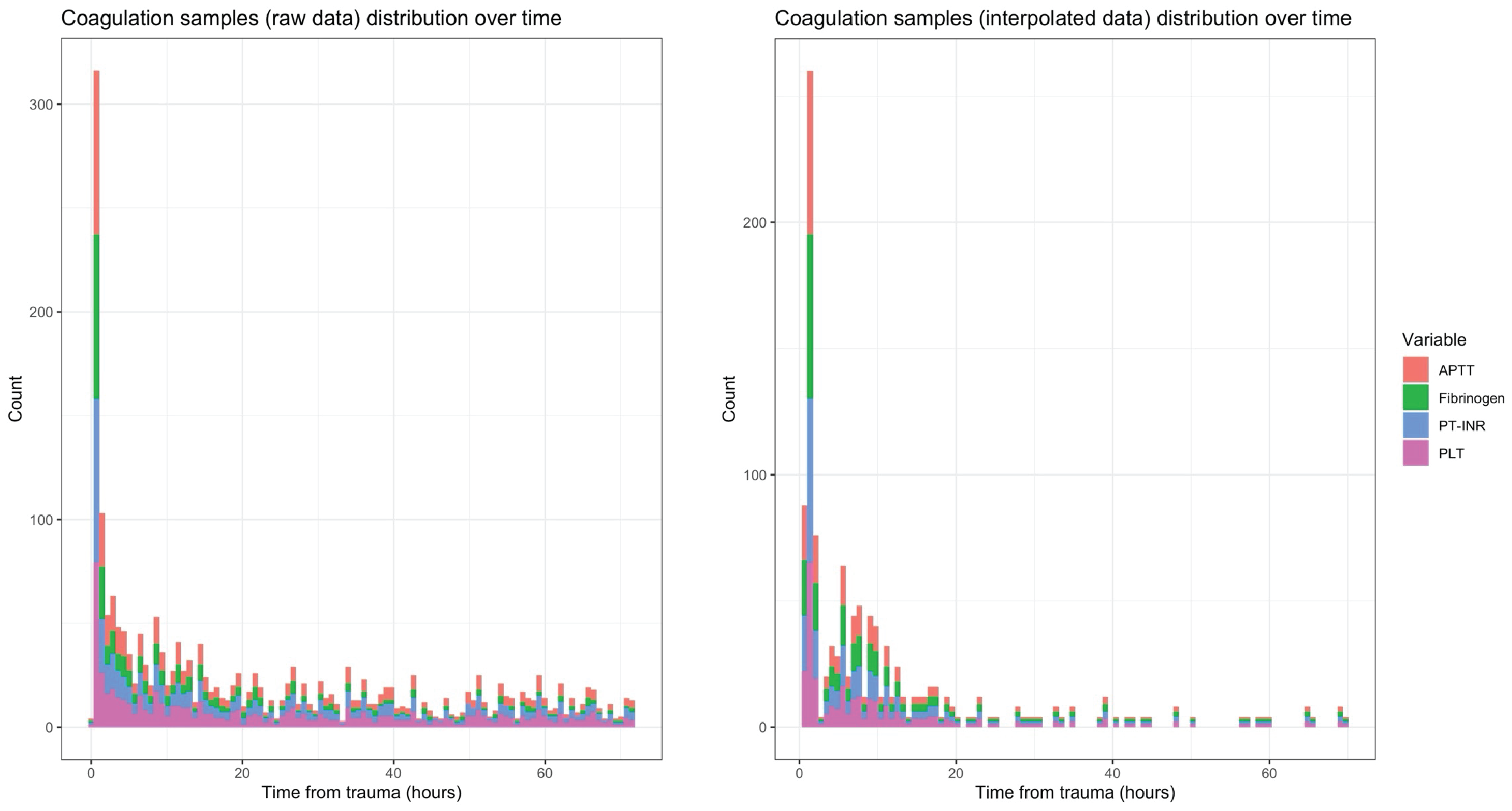
**

**Appendix C**: ACF/PACF of the Multivariate GAMM. The autocorrelation (ACF) and partial autocorrelation (PACF) functions were computed using the acf and pacf functions from the stats package. The plots show a slight cyclic time trend, which was acknowledged but not incorporated into the current analysis.
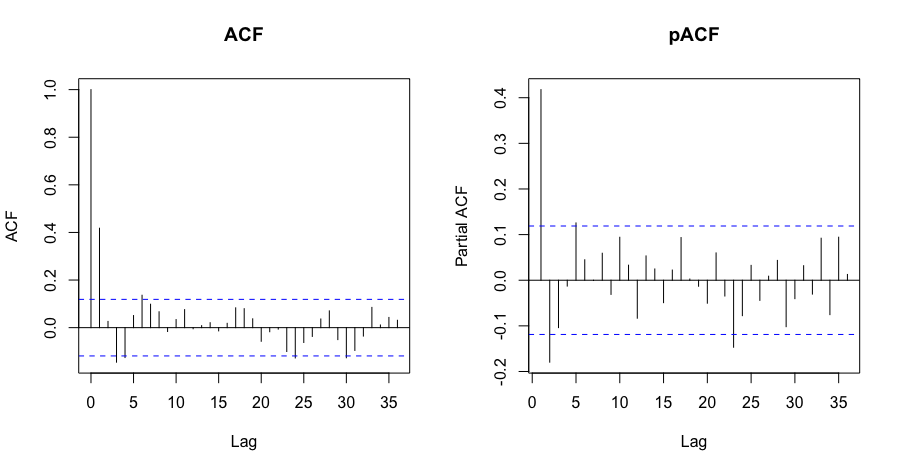


**Appendix D**: Correlation matrix of APTT, PT-INR, PLT, fibrinogen, sex, age, time from trauma, GCS, pupil response, and contusion volume. Correlations are displayed numerically and by color intensity; crossed-out values are not statistically significant. Abbreviations: APTT = activated partial thromboplastin time; GCS = Glasgow Coma Scale; INR = international normalized ratio; ml = milliliters; PLT = platelet count; PT = prothrombin time.


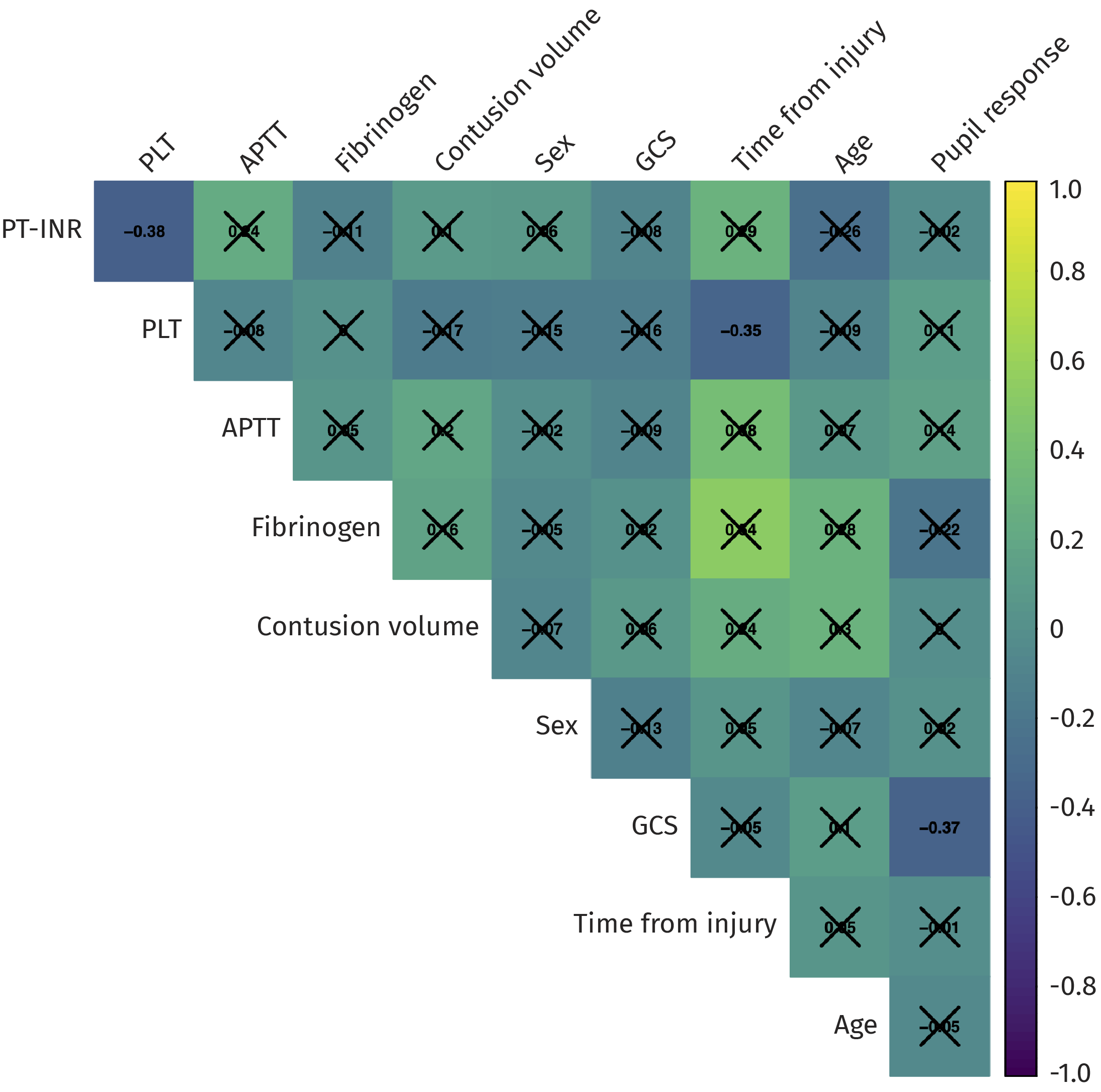

Supplement: Supplementary file 1 — Supplementary file1 (DOCX 2588 KB) [file 701_2026_6768_MOESM1_ESM.docx]
